# An Unusual Case of Recurrent Hypersensitivity Reaction Associated with Kounis-Like Acute Coronary Syndrome

**DOI:** 10.1155/2017/6421208

**Published:** 2017-08-27

**Authors:** Shanker Kundumadam, Vivek Reddy, Sagar Mallikethi Reddy, Pradeep Kathi, Aiden Abidov

**Affiliations:** ^1^Wayne State University School of Medicine, Detroit, MI, USA; ^2^Cardiology, John D. Dingell VA Medical Center, Wayne State University, Detroit, MI, USA

## Abstract

There have been multiple reports of allergic reactions associated with acute coronary syndromes. This has been classically described as Kounis syndrome. We present an unusual case of 70-year-old male with multiple prior hypersensitivity reactions and history of coronary artery bypass grafting who presented recurrent episode of severe angioedema and anaphylaxis. He responded to epinephrine but subsequently developed a non-ST elevation myocardial infarction with worsening heart failure. Our case is unique in that, unlike classic Kounis syndrome, the acute coronary event in this case did not present concurrently with the allergic reaction; rather it took nearly 48 hours to present. Subsequent angiogram revealed patent grafts and significant decline in the left ventricular systolic function as compared to his own ECHO a year ago. We postulate that slow mediators of inflammation may play a role in delayed development of acute coronary events with associated LV dysfunction following episodes of angioedema and anaphylaxis.

## 1. Introduction 

We present a 70-year-old male with known coronary artery disease (CAD) and multiple episodes of recurrent angioedema and anaphylaxis. During his last admission, the patient developed a non-ST elevation myocardial infarction with worsening left ventricular systolic dysfunction within 24–48 hours of admission. The angiogram revealed patent coronary grafts.

## 2. Case

**Figure 1:**
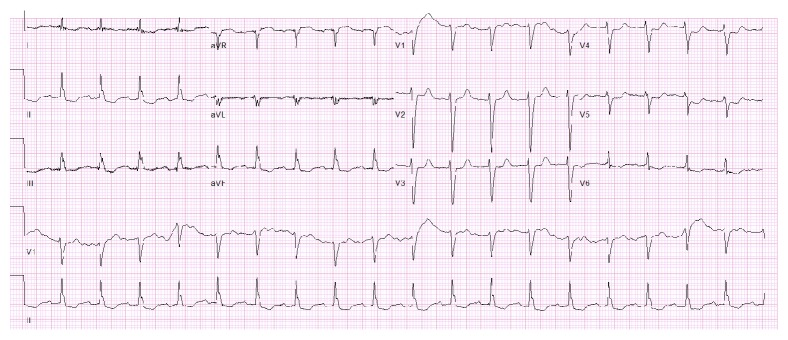
EKG on presentation reveals normal sinus rhythm with heart rate of 108 bpm.

A 70-year-old male with a past medical history significant for recurrent angioedema and anaphylaxis with no known etiology, coronary artery disease (s/p coronary bypass graft surgery in 2007), and systolic congestive heart failure with a left ventricular ejection fraction (LVEF) of 35–40% presented to the hospital with an episode of severe angioedema and anaphylaxis. No specific trigger was identified for this event. Vitals on presentation were unremarkable with physical exam only significant for facial swelling and erythema. The remainder of the systemic examination was within normal limits. His initial EKG on presentation is shown in Figure 1. For the treatment of anaphylaxis, he received intramuscular epinephrine (with sodium metabisulfite preservative) in the ER to which he responded well. Of note, his previous episodes of hypersensitivity reactions were also treated with epinephrine with similar composition. He also received antihistamines and prednisone along with his home medications as aspirin, carvedilol, atorvastatin, and furosemide.

**Figure 2:**
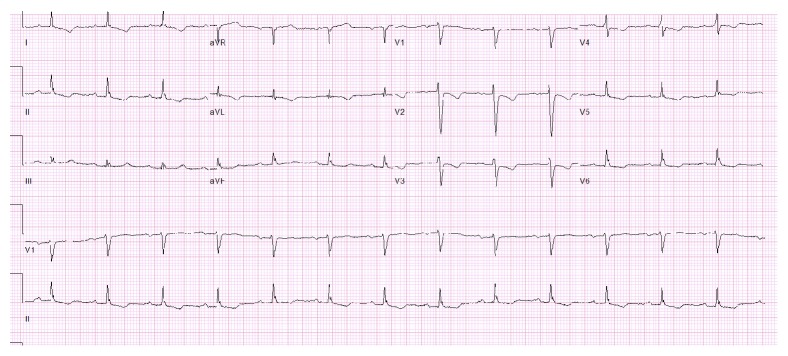
EKG shows normal sinus rhythm with heart of 86 bpm and new T inversions in V1 to V4.

**Figure 3:**
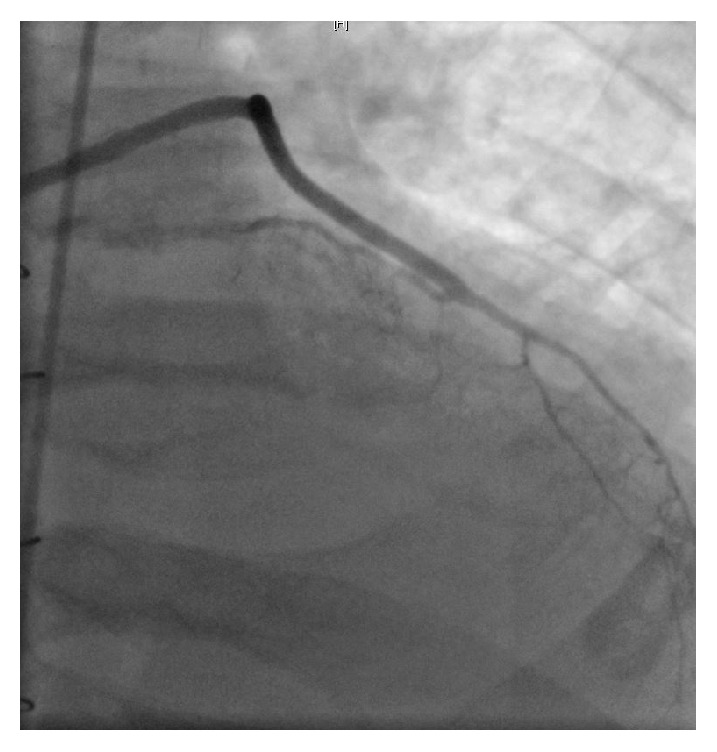
Angiographic view of the patent saphenous venous graft to obtuse marginal branch.

**Figure 4:**
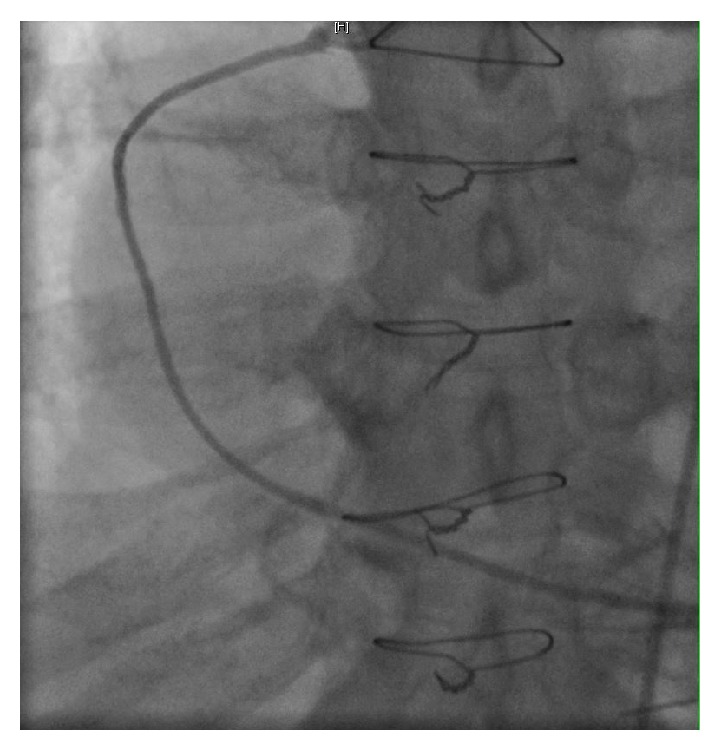
Patent saphenous venous graft to posterior descending artery.

**Figure 5:**
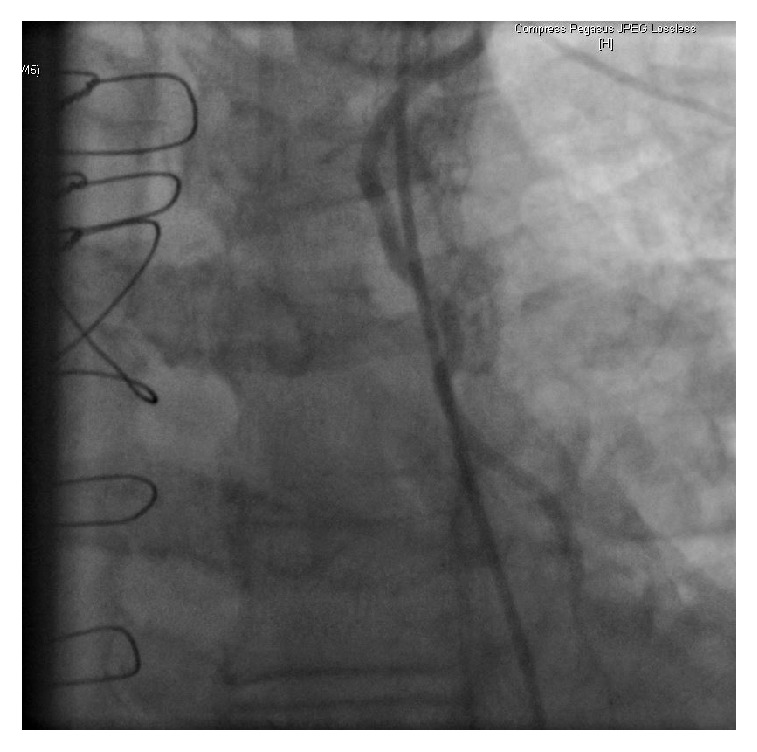
Interpretation: Patent LIMA graft to LAD.

On Day 2 of his hospitalization, the patient developed chest discomfort along with worsening of shortness of breath. His troponin U trended up from normal range on presentation to 8 ng/ml (normal < 0.80). The follow-up EKG is shown in Figure 2. The non-ST elevation myocardial infarction (NSTEMI) clinical protocol was initiated and the patient underwent a coronary angiogram (Figures 3–5). Of note, the patient did not have any significant tachycardia after the epinephrine administration.

The angiogram revealed severe native vessel CAD, patent coronary grafts with a calculated LVEF during the left ventriculogram estimated at 15%, a significant decline from his baseline LVEF of 35–40% a year ago. The patient was medically managed for systolic congestive heart failure and non-ST elevation myocardial infarction, with clinical improvement. Allergy and immunology workup to identify particular triggers for the hypersensitivity reactions were negative. Workup on complement enzyme defects was also negative. The patient was later discharged home on appropriate medications with outpatient cardiology and allergy-immunology follow-up.

## 3. Discussion

There have been multiple reports of allergic reactions associated with acute coronary syndromes. This has been classically described as Kounis syndrome, which was first published in 1991 [1, 2]. There have been 3 types of Kounis syndrome described: vasospastic anginal form, known preexisting coronary atheromatous disease, and that associated with stent thrombosis [3, 4]. The vast array of cytokines released from mast cell degranulation, along with histamine, neutral proteases, tryptase, chymase, arachidonic acid products and platelet-activating growth factor have been postulated as the mediators responsible for these coronary events [1, 2, 5]. Most of the cases described in this context report acute coronary events, which happen alongside the allergic or anaphylactoid reaction. Our case is unique in that, unlike classic Kounis syndrome, the acute coronary event in this case did not present concurrently with the allergic reaction; rather it took nearly 48 hours to present. This finding implies that many other mediators, especially slow mediators of inflammation, may play a role in delayed development of acute coronary events along with left ventricular systolic dysfunction during episodes of angioedema and anaphylaxis.

Treatments of such allergic reactions associated with acute coronary events necessitate addressing both these parts individually. This may pose a clinical challenge as many medications involved in treating acute coronary syndrome such as morphine, aspirin, and heparin can by itself trigger mast cell degranulation and precipitate coronary events. Epinephrine, used to treat allergic reactions, could also precipitate coronary events, which demands its cautious use especially in a patient with preexisting CAD. It is worthwhile to check serial troponins on these patients presenting with severe allergic reactions [6].

## 4. Conclusion

The association of anaphylaxis, angioedema, and other allergic reactions with acute coronary syndromes is well documented and represents an entity known as Kounis syndrome. While three variants of this syndrome exist, our case exemplifies that there may be other previously undefined pathophysiologic mechanisms present. We describe a case of anaphylaxis and angioedema with delayed coronary event findings suggestive of the possibility of a late inflammatory response resulting in myocardial injury. To our knowledge, no previous documentation of this variant exists, warranting further research and studies in the pathophysiology of acute coronary syndromes secondary to such an event, particularly with focus on delayed mediators of inflammation.
